# Effectiveness of iconic therapy for the reduction of borderline personality disorder symptoms among suicidal youth: study protocol for a randomised controlled trial

**DOI:** 10.1186/s12888-018-1857-x

**Published:** 2018-09-03

**Authors:** Silvia Hurtado-Santiago, José Guzmán-Parra, Rosa M. Bersabé, Fermín Mayoral

**Affiliations:** 1 Saint John of God Psychiatric Centre, Hospitaller Order of Saint John of God, Málaga, Spain; 2 Mental Health Department, Medical Research Institute (IBIMA) of Málaga, Regional University Hospital of Málaga, Málaga, Spain; 3 Department of Psychobiology and Methodology of the Behavioural Sciences Department, University of Málaga, Málaga, Spain

**Keywords:** Iconic therapy, Borderline personality disorder, Psychological therapy, Personality disorders

## Abstract

**Background:**

Borderline personality disorder (BPD) is associated with an intensive use of mental health services, even in the absence of a full diagnosis. Early symptom detection and intervention may help alleviate adverse long-term outcomes. Iconic Therapy is an innovative manual-driven psychotherapy that treats BPD symptoms in a specific and intensive manner. Preliminary results are promising and the indication is that Iconic Therapy may be effective in reducing BPD symptoms. The aim of this study is to assess how effective Iconic Therapy is compared to Structured Support Therapy in a real clinical setting.

**Methods/Design:**

Our study will be a controlled 12-month pragmatic, two-armed RCT. A total of 72 young people (15 to 25 years old) with suicidal ideation/self-injuring behaviour and BPD traits and symptoms will participate in the study. The subjects will be randomised into two groups: Iconic Therapy or Structured Support Therapy. The participants will be assigned to either group on a 1:1 basis. Both the Iconic Therapy and the Structured Support Therapy programmes consist of 11 weekly sessions delivered by two trained psychologists in a group format of between 8 to 12 outpatients. The primary outcome will be measured by the change in symptom severity. Secondary outcomes include changes in suicidal ideation/ behaviour, non-suicidal self-injury, maladjustment to daily life and cost-effective analysis. The primary outcome will be a decrease in the severity of BPD symptoms as assessed by the Borderline Symptom List (BSL-23). For the clinical evaluation, three study assessments will take place: at baseline, after treatment and at 12-month follow-up. We hypothesise that patients attending the Iconic Therapy group will show a significantly higher reduction in symptoms than those in the Structured Support Therapy group. Data will be analysed using generalised estimating equation (GEE) models.

**Discussion:**

By responding to the need for briefer and more comprehensive therapies for BPD, we foresee that Iconic Therapy may provide an alternative treatment whose specific therapeutic principles, visually represented on icons, will overcome classical Structured Support Therapy at reducing BPD symptoms.

**Trial registration:**

NCT03011190

## Background

The characteristics of Borderline Personality Disorder (BPD) are dysregulation of emotional, behavioural and interpersonal relations [1]. BPD is very prevalent among teens and young adults, but diagnosis is often delayed and early treatment rarely offered [2]. Studies show that, with a diagnosis rate ranging from 0.5 to 31.2% among this young population [3], BPD is a disorder with a high social and economic impact, of which the most severe consequence is suicide.

Emotional dysregulation, or mood instability, has been defined as rapid oscillations of an intense affect with a difficulty in regulating said oscillations or their behavioural consequences [4] and is a key factor in the development and maintenance of BPD [5]. Associated with emotional dysregulation, suicidal ideation and behaviour is another psychiatric problem that usually occurs [6, 7]. Severe cases of emotional dysregulation approaching the criteria for BPD diagnosis have a global suicide rate of 8% [8]. An important risk factor for suicide is self-injury behaviour [9], which is considered as an intentional, self-directional act to harm oneself [10]. It is estimated that around 50% of suicidal adolescents have had previous self-injury behaviour [11]. One of the reasons adolescents show affinity to self-injury seems to be their lack of ability to regulate emotions [12, 13]. In fact, self-injury is considered a maladaptive coping mechanism rather than an intentional act to die [14], despite the fact that suicide may result in death for the habitual self-injurer [15]. Nevertheless, personality traits are more malleable and sensitive to change during adolescence [16, 17] and for that reason, early detection and intervention of self-injury behaviour is a unique opportunity to prevent an aggravation of emotional instability, BPD diagnosis and suicidal acts.

To date, the majority of studies have recommended psychotherapy as the main treatment for people with BPD [18], but in clinical practice, pharmacotherapy is the primary treatment, even though the benefits are unclear [19, 20]. Several manualised psychotherapies for BPD have proven their beneficial effects to date [21]. One recent meta-analysis [22] suggests that Dialectical Behaviour Therapy (DBT) [23] and psychodynamic psychotherapies (e.g., Mentalization-Based Therapy (MBT) [24] and Transference-Focused Therapy (TFT) [25]) are more effective than non-specialised psychotherapies or treatment as usual (TAU). Other psychotherapies, such as Schema-Focused Cognitive Therapy (SFT) [26] and STEPPS (Systems Training for Emotional Predictability and Problem Solving) [27], have also shown effectiveness in the treatment of BPD. Regarding evidence-based treatments for self-injury behaviour, very few studies exist to date [28–30].

From a general scientific setting, recommendations to develop innovative therapies by combining and adapting current treatments have been made [2, 31–36]. Iconic Therapy is a comprehensive psychological intervention that integrates existing therapeutic principles proven to be effective in educating people who have difficulty regulating their emotions. What makes Iconic Therapy different from many other approaches is the use of images to help those affected by BPD understand the origins and mechanisms of perpetuation of their emotional instability, feel validated and acquire the necessary attitudes and skills for daily life during a 12-week intensive programme. These images (a total of 35) include pictures, drawings and coloured geometric figures of neutral emotional valence (such as a table, a sailboat or a tower of books) that symbolically represent therapeutic principles (e.g., acceptance, resilience or empathy). The group of participants visualise from 2 to 5 icons per session on a big screen as the therapist explains their therapeutic symbolism, which seemingly eases a better understanding and more rapid evocation of therapeutic clues in crisis moments. Iconic Therapy was created in 2004 by the psychologist Soledad Santiago and first trialled in a personality disorder unit at a psychiatric hospital in Málaga. It has also been used in other locations across Spain, supported by a text book (Iconic Therapy) [37]. Guidelines for Iconic Therapy have been prepared and are currently pending publication. It contains examples and a number of therapists’ considerations.

Iconic Therapy is a two-part programme composed of a) an intensive 12-week programme and b) an additional 1- to 2-year programme. The intensive programme duration is typically 10–12 weeks, but can vary among users depending on the group’s particular motivation and participation. Therapy is administered to patients as a group and includes weekly sessions led by a therapist and co-therapist in a classroom setting similar to a psychoeducative seminar or workshop. Each group consists of 8 to 12 participants on an outpatient basis. At the end of each and every group session, the Iconic Therapy materials are handed out so participants can keep them as a resource during difficult periods. An additional number of 6 to 8 one-on-one sessions with the therapist are inserted every 2 sessions from the onset of the programme. During the second phase of the Iconic Therapy programme, an additional year of treatment will be offered. It will consist of 3–6 face-to-face sessions with the therapist that become less frequent over the duration of the course. Individual sessions from both parts of the Iconic Therapy programme are a key element; as during this time, patients are taught to select for themselves the best icon for every situation, thus learning new behavioural skills in the relevant context. Iconic Therapy is an intensive treatment to be delivered alone or as a complement to their usual treatment, depending on the history and severity of the symptoms.

When considering the effectiveness of Iconic Therapy on BPD symptoms, preliminary studies have shown good clinical results, including a decrease in functional impairments [38] and assertive behaviour amelioration [37, 38]. The first preliminary study [38] took place on 7 BPD diagnosed patients in the context of a psychiatric hospital ward and showed a significant improvement in emotional dysregulation, relationship instability and impulsivity as well as increased assertive behaviour (familiar, academic and social) 2 years after receiving Iconic Therapy. The second study [39] followed 4 outpatient adolescents attending a public mental health service and having 2 or more BPD DSM-IV-TR criteria. It showed a non-significant improvement in emotional dysregulation, relationship instability and impulsivity as well as equally non-significant assertive behaviour achievements after treatment, which may be explained by the small sample size. Still remarkable is the high adherence and null dropout rate, suggesting good treatment acceptance by patients. Despite the promising results, it is important not to overlook the fact that both of these preliminary studies lack a control group, which importantly limits their relevance.

This clinical trial is aimed at overcoming this limitation by comparing two groups: Iconic Therapy and Structured Support Therapy. It is hypothesised that participants receiving Iconic Therapy will demonstrate greater improvement in severity of BPD symptoms after treatment and at 12-month follow-up compared to those receiving Structured Support Therapy. As a secondary hypothesis, impairment in adaptive functioning (suicidal ideation, suicidal behaviour, maladjustment to daily life and use of services) is expected to improve in parallel.

## Methods

### Study design

The study is planned as a 12-month pragmatic, two-armed parallel randomised controlled trial (RCT). It will randomise 72 young subjects (aged between 15 and 25) with suicidal ideation and/or self-injuring behaviour and borderline personality traits. Participants will be randomly assigned to one of two groups, Iconic Therapy or Structured Support Therapy, on a 1:1 basis to form 4 groups of 18 patients. Two of these groups will be included in the Iconic Therapy group and the other two in the Structured Support Therapy group. A total of 8 volunteer psychologists will administer the therapies formed into 4 pairs of therapists and co-therapists. The volunteer psychologists are required to have obtained a Master’s Degree in Psychology and have previous experience with patients. It is inherently not possible to blind psychologists delivering the Iconic Therapy intervention, since they have been specifically trained for 10 months. The goal of the psychologists conducting the control group is to obtain good clinical results. They will achieve this through therapeutic intervention.

Primary and secondary outcome measures will be assessed at baseline, after treatment (10–12 weekly sessions) and at 12-month follow-up (15 months after the baseline). Study variables and assessment times are shown in Table 1.

**Table 1:** Study variables and assessment time

Instrument	Assessment area	Time of assessment
Primary outcomes		
BSL-23^a^	BPD symptoms severity	Baseline After treatment At 12 months
Secondary outcomes		
C-SSRS^b^	Suicidal ideation or behaviour	Baseline After treatment At 12 months
DSM-V criteria^c^	Non-suicidal self-injury diagnosis	Baseline After treatment At 12 months
CSRI^d^	Economic evaluation	Baseline At 12 months
Subsidiary measures		
IG^e^	Daily life adjustment	Baseline After treatment At 12 months
CEQ^f^	Credibility/expectancy questionnaire	After treatment At 12 months
Ad hoc scales^g^	Perceived subjective global improvement *(self-reported)*	After treatment At 12 months
Ad hoc scales^h^	Perceived global improvement *(family/friends reported)*	After treatment At 12 months

Notes. BSL-23^a^ (Borderline Symptom List- short form); C-SSRS^b^ (Columbia Suicide History Form); DSM-V criteria^c^ (Non-suicidal self-injure diagnosis according to the Diagnosis and Statistical Manual of Mental Disorders, DSM- V); CSRI^d^ (Client Service Receipt Inventory); IG^e^ (maladjustment scale)**;** CEQ^f^ (credibility/expectancy questionnaire); Ad hoc scales^g^ (Perceived subjective global improvement); Ad hoc scales^h^ (Family and friends’ perceived global improvement)

**Fig. 1:**
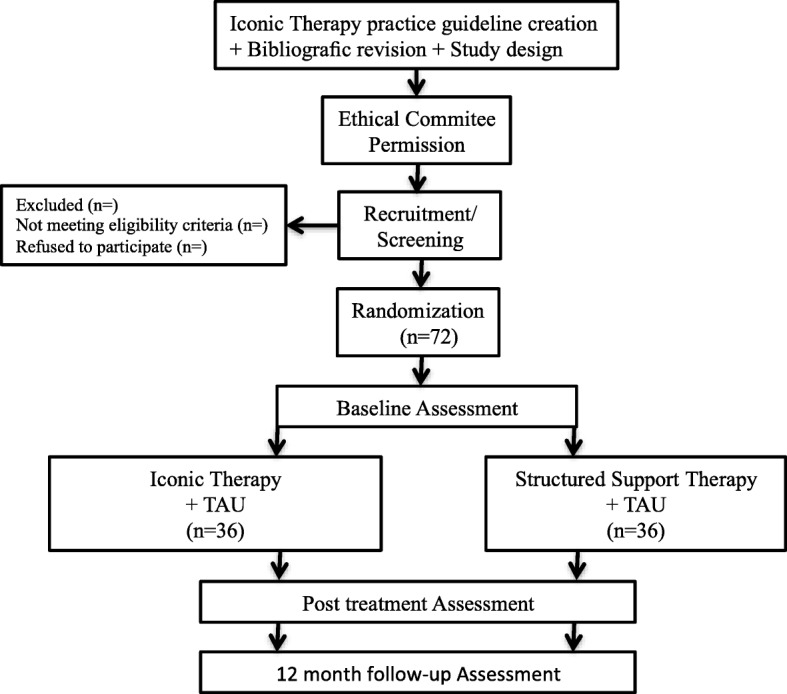
Study flowchart and chronogram. TAU: Treatment as usual

For ethical reasons, participants of the comparison group may attend Iconic Therapy once the study is completed. The study flowchart is shown in Fig. 1*.*

### Study aims

The aim of the study is to investigate, by comparison with Structured Support Therapy, how effective Iconic Therapy is in improving participants’ BPD symptoms. The primary outcome measure is a change of symptoms measured through the use of the Borderline Symptom List (BSL-23) post-treatment. Our hypothesis is that patients undergoing Iconic Therapy will show a greater reduction in the severity of their symptoms after receiving the intensive part of the therapy than those undergoing Structured Support Therapy. As a secondary outcome, we hypothesise that these differences will continue to be verifiable at the 12-month follow-up. Other secondary measures are suicidal ideation, suicidal behaviour, maladjustment to daily life and use of services as a consequence of BPD symptoms. We will also evaluate satisfaction with the treatment and perceived subjective global improvement beyond the symptoms.

### Participant eligibility

#### Inclusion criteria

The following are inclusion criteria for the study: a) be aged between 15 and 25, b) have suicidal ideation as detected when 2 or more from 5 categorical questions about suicidal ideation are affirmatively reported in the Columbia Suicide History Form (C-SSRS) [40], c) a score of ≥38 out of 84 on the Exploratory Questionnaire of Personality-III-BPD (CEPER-III-BPD) [41] and d) sufficiently proficient in Spanish to undergo treatment.

#### Exclusion criteria

Exclusion criteria includes the following: a) antisocial personality disorder as measured in the Clinical Interview for DSM-IV Axis II Disorders (SCID-II) [42], b) substance/alcohol abuse or dependence to the point that the therapist suspects it may interfere with their adherence to treatment, c) high suicidal risk as detected when suicidal ideation intensity is 4 or more over a 5-point scale as assessed by the C-SSRS [40] and d) a score of ≥35 out of 60 points on the Credibility/Expectancy Questionnaire (CEQ) [43], with the aim of considering if the subject expects to improve with treatment.

Efforts to reduce dropout rates will be made by providing support for travel arrangements and sending text message reminders to boost attendance. All participants will sign an informed consent form prior to inclusion in the study (parents must sign on behalf of participants under the age of 18). The study will follow the principles of Good Clinical Practice and the Declaration of Helsinki.

#### Setting and recruitment

Sample recruitment will take place at the Regional University Hospital of Málaga (Spain). This is a public mental health service with a catchment area of approximately 300,000 inhabitants. Psychologists and psychiatrists there will be given eligibility forms and will recruit outpatients until the required sample is filled.

### Study procedure

#### Screening assessment

Suicidal risk will be assessed by use of the C-SSRS [40]. Borderline personality or coexisting behavioural trends will also be measured with the CEPER-III-BPD [41]. Symptom severity will be measured through the BSL-23 [44].

#### Randomisation

Once grouped by age, the principal investigator will randomise patients into either the Iconic Therapy group or Structured Support Therapy group on a 1:1 basis.

#### Iconic therapy group

Iconic Therapy is a 12-week programme structured in 4 explicit manual-driven modules. Each module takes from 2 to 3 weekly group sessions with a duration of 90 min each: the first 30 min are to debate the practical use of last session’s icons and the next 60 to advance new ones. The programme content is focused on the essential impairments in personality functioning of BPD patients: intimacy/empathy, identity and self-direction as parts of a more general introductive module. A therapist and co-therapist will lead the group. Programme contents are summarised in Table 2.

**Table 2:** Summary of the contents of Iconic Therapy and Structured Support Therapy programmes

Therapy group	Programme composition^a^
Iconic therapy + treatment as usual^b^	Module 1: Understanding the problem, motivating and acquiring some basic coping skills. Session 1 *Content:* *-* Presentation. - Explicative model of pathology: visual understanding of the problem origins and perpetuation as an enduring vicious cycle (*the sinking, the blinding and the flight icons*) - Education to recognize patient’s affective reactions (*the map icon*). *Objectives:* Understanding that it is possible to develop healthier alternatives to replace dysfunctional patterns of thinking, feeling and behaving (to be learnt and trained during the length of the therapy). Session 2 *Content:* - Therapeutic model elaboration: emotional self-regulation techniques (*the key phrase and the cooling off time icons*). - Basic skills training: decision making and self-improvement techniques to manage remorse after hurting another (*the corsage of alternatives, the telephone and the zig-zag icons*). *Objectives:* Acquiring new learning experiences and general self-management skills to control inappropriate emotional responses. Module 2: Intimacy and empathy. Session 3 *Content:* - Interpersonal problems solution: expressing criticism to reach an agreement (*the sacks attack and the translator icons*). - Practical examples and role-playing in groups. *Objectives:* Experiencing self-effectiveness when sharing experiences in interpersonal relationships. Session 4 *Content:* - Interpersonal problems: accepting criticism and getting distance from negative relationships (*the black grain, the glass wall and the rock wall icons*). - Practical examples and role-playing. *Objectives:* Sharing experiences of interpersonal relationships in groups. Session 5 *Content:* Negative emotions and cognition management: - Hostility (*the mountain icon*) - Inhibition (*the pen icon*) - High expectations from others (*the bouquet of flowers icon*) - Cognitive filters (*the coloured sunglasses icon*) - External attribution (*the tower of books icon*) - Practical examples and exercises. *Objectives:* Detecting and changing negative ways of perceiving and interpreting other people or events. Practicing motivated behaviors, reflecting intentions and desires. Module 3: Self-esteem and identity. Session 6 *Content*: - Personality trait optimization (*the river icon*). - Others comparison avoidance (*the coloured bars icon*). - Overcoming the need to receive a torrent of affection (*the self-disqualification pitfall icon*). - Mimetization vs reaching a genuine position (*the mimetization and the positioning icons*). *Objectives:* Gaining a more stable sense of self and personal security. Session 7 *Content*: - Sense of Emptiness (*the Matryoshka dolls icon*). - Negative vs positive behavioral tendencies (*the positives and negatives icon*). - Prejudices avoidance (*the Mozart icon*). *Objectives:* Gaining a more stable sense of self and personal security. Session 8 *Content*: - Emotional balance clues (*the table icon*). - Personal dependence avoidance (*the flag icon*). - Uselessness of complaining about past experiences and how to take advantages of them (*the web and the embankment phrase icons*). - Practical review by sharing practical uses of the icons on group. *Objectives:* Reaching independent self-advise on daily situations. Module 4: Self-direction. Session 9 *Content:* - Liberty vs impulse slavery: reconceptualization (*the bypass road icon*). - Goals setting (*the range icon*). *Objectives:* Strengthening impulsiveness and risk-taking control. Session 10 *Content:* - Adversity as an impulse towards the goal (*the sailboat icon*). - Values and prosocial standards to guide our decisions (*the compass icon*). - Practical exercises of people finding their own concretes and vital goals. *Objectives:* Instigating normative ethical behaviour. Final review (Session 11): Relapse prevention. Workshop reviewing learned strategies and post-treatment evaluation. Sixth month review (Session 12): Relapse prevention. Workshop reviewing learnt strategies. Twelveth month review (Session 13): Relapse prevention. Workshop reviewing learnt strategies and 12-month follow-up evaluation.
Structured support therapy + treatment as usual^c^	Session 1: Social relationships. *Content:* - Presentation. - Video and debate. May friends *or* acquaintances turn into relatives for you? / What is the importance of having a friend to stand by? / Is it possible to find a friend on the internet?. *Objectives:* Participants interacting with other participants of similar characteristics in a casual and secure environment. Session 2: Risks on the internet. *Content:* Video and debate. What do you use social websites for? selfies, finding partners, photos, risks, problems. *Objectives:* Participants interacting with other participants of similar characteristics in a casual and secure environment. Session 3: Emotional instability and impulse control. *Content:* - Information about the role of emotion regulation and emotional instability (coordinate axis) in emotional disorders. - Impulsivity definition. The turtle technique (detect the emotion, stop, hide and breathe in the shell, go out and think of a solution). *Objectives:* Debating about the given topics and sharing strategies to deal with them. Session 4: Emotional regulation and jacobson relaxation technique. *Content:* - Role-playing: The therapist reads a short story and the participants play a role simulating different attitudes towards the story (distracted, aggressive or interested). The rest of the participants try to detect name, intensity and coping skills to deal with shown emotions. - Soft music and Jacobson relaxation technique. *Objectives:* Session 5: Social skills. *Content:* - Defining empathy and assertiveness. - Role-playing “the assertiveness umbrella”: the participant with the umbrella is required to express his complaints in front of the rest of the group. *Objectives:* Learn to detect internal and external social skills. Session 6: Social skills ii. *Content:* - Role-playing. The participants are invited to introduce themselves, ask the therapists questions, maintain a conversation with their closer partner or express, in his/her opinion, the best characteristic of the partner on the left. *Objectives:* Increasing assertive behavior in a secure environment. Session 7: Self-image and communicational styles. *Content:* - Defining identity and self-image by thinking of an admired person (famous, family or friends) and detecting similar traits within themselves. - Defining communicational styles: passive, aggressive, assertive. - Videos with examples of people with different communicational styles. *Objectives:* Learning to detect their own and other’s necessities. Session 8: Mindfulness. *Content:* - A specialized volunteer psychologist delivers a mindfulness session lying on the floor, with raisins to be consumed by the therapist and participants as a shared contemplative experience. *Objectives:* Enhancing stress tolerance and contemplation capacity. Session 9: Self-esteem. *Content:* - Defining self-esteem and main personal characteristics: each participant writes his or her 5 main characteristics on an anonymous paper and gives it to the therapist, who reads them all. When there is a negative characteristic, the therapist tries to explain its usefulness and the way to make it positive. *Objectives:* Improving self-awareness. Session 10: Self-esteem ii. *Content:* - Self-esteem: participants are incited to exercise their body, trust in their qualities and use them, don’t look for other’s approval, take perspective and accept their problem. - Self- realization: bearing in mind their best qualities, participants are invited to think of an activity they are motivated for as well as different ways to keep in touch with it. *Objectives:* Improving the self-esteem. Session 11: Relapse prevention. Reviewing the strategies learned across the program and contents and post treatment evaluation. Sixth month review (Session 12): Relapse prevention. Workshop reviewing learnt strategies. Twelveth month review (Session 13): Relapse prevention. Workshop reviewing learnt strategies and 12-month follow-up evaluation.

Notes. ^a^All patients are free to contact the principal investigator throughout the process to solve practical questions about the use of session contents. ^b^10–12 group sessions given by 2 specifically trained psychologists using the guideline draft. ^c^11 group sessions given by 2 psychologists using a non-manualized program

A total of 3 more group sessions (2 of them matched with the assessment) will be delivered by the therapist and co-therapist after treatment and at 6 and at 12 months follow-up as well as an undetermined number of face-to-face sessions with the principal investigator. The same is true for the Structured Support Therapy group. These sessions are aimed to assure participants’ needs are met and to minimise dropout rates in both groups. Nevertheless, the Iconic Therapy group will continue to see and reinforce visual images, whereas those in the control group will receive plain verbal explanations without illustrations.

As this is the first time the Iconic Therapy programme is being delivered by therapists other than its creator or principal investigator, group sessions will be supervised from behind a unidirectional window by the principal investigator. Also, two Iconic Therapy randomised group sessions will be recorded by the creator for analysis. During the 10–12 week period of the study, supervision and feedback will be given to the therapist and co-therapist of both study groups.

#### Structured support therapy group

Participants will take part in a 60-min weekly group session for 10–12 weeks. Sessions will be 30 min shorter than those for the Iconic Therapy group, as verbal explanations do not require further practical clarifications by therapists and a longer duration could affect the adherence. For the second part of the programme, 3 group sessions and a variable number of individual face-to-face sessions will be offered to this group, same as for the Iconic Therapy group. The aim of face-to-face sessions is to meet the needs of participants and to provide support throughout the study. The contents of the programme can be seen in Table 2.

#### Treatment as usual

Patients in the trial will continue their ambulatory treatment from public specialised mental health services as usual. TAU may include medication with weekly or monthly individual sessions with the referring psychologist.

#### Therapy attendance and compliance

At the 12-month follow-up, the trial for both groups will have concluded. A participant has to attend a minimum of 8 group sessions to be considered as having completed treatment.

#### Data collection and outcome measures

Table 1 displays the outcome measures and corresponding instruments and assessment times. Patients will be assessed at baseline, after treatment and at 12-month follow-up.

#### Primary outcome measure

##### Severity of borderline personality disorder

We will use the Spanish version of the BSL-23 [44]; it replicates the one factor structure of the original and shows high reliability (*α* = .95) as well as good test-retest stability when checked in a subsample of 74 patients (*r* = .73 *p* < .01). The BSL-23 [45] is one of the most widely used questionnaires (in its short form) in psychotherapy trials. It has 23 self-answer questions rated on a scale of 1 to 5 on the Likert scale, ranging from 0 (not at all) to 4 (very strong). The BSL-23 is very reliable in the diagnosis of BPD, and as evidenced by Cronbach’s alpha of .93, its one factor structure makes it highly constant.

#### Secondary outcome measure

##### Suicidal ideation and behaviour

The C-SSRS [40] is a validated tool for quantifying the existence or severity of suicidal ideation and behaviour. It has 2 sub-scales to assess ideation and behaviour separately. Ideation severity is assessed on a 5-point Likert scale within a range of 1–5. The existence of suicidal behaviour is assessed in the same manner. This ranges from 0 (very little/no physical harm) to 4 (intensive care service required). The C-SSRS demonstrates good convergent and divergent validity. Also, it displays high sensitivity and specificity for the classification of suicidal behaviour. We will provide a self-report form for the questionnaire.

##### Non-suicidal self-injury criteria

In this study, criteria from the DSM-V [1] regarding self-injury is used to assess participants.

##### Economic evaluation

The Client Service Receipt Inventory (CSRI) [46] measures the use of healthcare/social care services and other economic impacts. This questionnaire measures direct costs (emergency services/hospital admissions use, specialised medical consultations, prescribed diagnostic trials and consumed medication) and indirect costs (absenteeism and quantity/quality of job performance on a 100-point scale). Delivery will be by the use of an adapted Spanish version [47] in a self-reporting format.

#### Subsidiary measures

##### Maladjustment to daily life scale

The Maladjustment Scale (IG) [48] is used to assess the degree to which emotional dysregulation affects daily life functioning in psychiatric patients. It is a 6-item self-report questionnaire rated using the 6-point Likert scale, ranging from 0 (not at all) to 5 (very strong). This test has a cut-off point of 12 for the full scale and 2 points for each item. The higher score, the greater maladjustment. It has a high internal consistency (α = .94) and high diagnostic efficiency (90%), showing sensitivity to therapeutic changes.

##### Credibility/expectancy questionnaire

Satisfaction with treatment is assessed by the CEQ [43]. It is a brief 6-item questionnaire covering how logical the treatment seemed, the extent of patient satisfaction, whether other psychological problems could benefit from it, its usefulness for the patient’s specific problem and to what extent the treatment was aversive, rated on a scale from 0 (not at all) to 10 (very strong). This instrument has demonstrated high internal consistency (α = .79 for expectancy and α = .81 for credibility) and good test-retest reliability (*r* = .82 for expectancy and *r* = .75 for credibility) [49]. The questionnaire is delivered post-treatment and at 15 months after the baseline.

##### Perceived subjective global improvement

Participants will be asked at the end of the study on a 7-point ad hoc Likert scale from much/quite/little bit better, the same or much/quite/little bit worse compared to when they entered the study. Since BPD patients usually have a distorted perception about themselves, the same scale will be used to closely evaluate the perceived impressions of a family member or friend.

#### Baseline variables

##### Socio-demographic variables

Gender, age, marital status, parent’s nationality, cohabitation, educational level and occupational status will be included in the study.

#### Study integrity

This study has been approved by the Provincial Ethics Committee of Málaga (Spain). The study has been developed in accordance with CONSORT guidelines.

#### Sample size

The sample size calculation is based on our primary outcome measure, BPD symptom severity at 12 months. A sample size of 26 participants per group is required to detect a post-treatment effect size of 0.20 (Cohen’s d) between both groups with a power of 0.80 in a one-tailed test and with a 95% confidence level. This corresponds to the estimated differences between groups of about 8.6 BSL-23 raw scores. Therefore, the total sample size is determined at 52 participants. Dropout rates for BPD patients are 24% to 52% [50]; therefore, we need to recruit at least 72 individuals.

#### Data analyses

Patients will be included in intent-to-treat analyses. Participants’ demographic characteristics will be summarised using descriptive statistics, and the comparability of the treatment groups will be examined using *χ*^2^ tests for categorical variables and *t* tests for continuous variables.

Data analyses of the primary and secondary outcomes will be conducted using generalised estimating equation (GEE) models. Essentially, GEE models extend generalised linear models (GLMs) to allow analysis of repeated measurements or other correlated observations. The response can be scale, counts, binary or events-in-trials. In addition, GEE models can also deal with data that have missing values as long as the missing value is completely random. Parameter estimation can still yield robust results [51].

In these GEE models, treatment conditions (experimental vs. control) will be included as the between-subjects factor and time (baseline, after treatment and at 12 month follow-up) as the repeated measure. Main effects and interaction between treatment and time will be examined. When the interaction effect is statistically significant, simple main effects analyses will be conducted.

IBM SPSS (version 22.0 for Windows) will be used for the statistical data analyses. Statistical significance will be defined as *p* < .05. Bonferroni correction will be applied for post hoc multiple comparisons.

## Discussion

BPD is a prevalent disorder with high functional impairment and health resources usage, of which the diagnosis and treatment are rarely offered at an early stage. Further alternatives to treat BPD and more studies comparing their effects are needed [2, 17, 22, 31, 32, 52], especially in adolescents and young adults [2, 17, 53].

A number of highly specialised psychotherapies have proven to be effective in BPD treatment. The strongest evidence favours DBT and psychodynamic approaches, but a major limitation is long duration and complexity. For example, the standard model of DBT is rarely completed in a real-life clinical setting [11]. Several dismantled versions of DBT approaches, such as DBT individual therapy without DBT skills training or DBT skills training without skills coaching, have also obtained good clinical results in BPD treatment [22, 52].

This study represents the first clinical trial to test the usefulness of Iconic Therapy, which may represent an alternative treatment of theoretical and practical value, due to its brevity together with its intensity and easy transport for scaling out across services for specialised early intervention services for BPD in the mental health system.

Negative emotional experiences are known to especially alter the capacity of BPD patients to recover cognitive aspects and task performance. The use of images makes mental representations last longer [54–56], especially when associated with emotionally relevant experiences [57, 58]. Iconic Therapy may represent a single therapy to reduce the frequency and intensity of negative emotions beyond BPD by using generic images to represent therapeutic aspects for patients to connect with their own autographical memories. Added to this, international use is possible, as the images used are generic.

We hypothesise that the use of the Iconic Therapy programme will enhance the awareness of BPD patients and their capacity to manage difficult situations, resulting in the further improvement of BPD symptom recovery. Here we describe the rationale, design and methods of the Iconic Therapy programme.

Specific therapies of this type may help sustain BPD sub-threshold symptoms and decrease health service and non-health care related costs derived from BPD diagnosis. Validating a treatment that has been showing good clinical results on severe BPD symptoms is not only an encouraging point of departure but also a priority. In this study, we test Iconic Therapy against Structured Support Therapy. We did not compare Iconic Therapy with any specialised treatment for BPD (i.e., DBT, TFT or MBT), as our objective was to empirically support that Iconic Therapy is more effective in reducing BPD symptoms than non-specialised treatment.

Finally, a number of potential limitations should be indicated. First, the sample size is small, and this may alienate results from its generalisation. Second, the age range for recruitment is limited (15 to 25 years old); therefore, the recruitment flow may be delayed. Third, heterogeneity on suicidal ideation severity and self-injuring behaviour frequency/lethality may affect treatment adherence, although symptom severity does not seemingly affect adherence to prescriptive therapies [59]. Fourth, the expected rate of dropouts for BPD is high (24% to 58%), and a high dropout rate may slant results [50]. Fifth, the 30 min shorter duration of the Structured Support Therapy group might interfere with the placebo control effect. Sixth, we used self-report questionnaires and not structured clinical interviews, which could represent a limitation despite the adequate psychometric properties of the instruments used.

## Abbreviations

BPD: Borderline personality disorder
BSL-23: Borderline Symptom List
CEPER-III-BPD: Exploratory Questionnaire of Personality-III-BPD
CEQ: Credibility/ Expectancy Questionnaire
CONSORT: Consolidated Standards of Reporting Trials
CSRI: Client Service Receipt Inventory
C-SSRS: Columbia Suicide History Form
DBT: Dialectical Behaviour Therapy
DSM-IV-TR: Diagnostic and Statistical Manual of Mental Disorders, Fourth Edition
DSM-V: Diagnostic and Statistical Manual of Mental Disorders, Fifth Edition
GCP: Good Clinical Practice
GEE: Generalised Estimating Equations
GLM: Generalised linear model
IG: Maladjustment Scale
MBT: Mentalization-Based Therapy
RCT: Randomised Controlled Trial
SCID-II: Clinical Interview for DSM-IV Axis II Disorders
SFT: Schema-Focused Cognitive Therapy
STEPPS: Systems Training for Emotional Predictability and Problem Solving
TAU: Treatment as Usual
TFT: Transference-Focused Therapy
